# Identification of candidate cancer predisposing variants by performing whole-exome sequencing on index patients from *BRCA1* and *BRCA2*-negative breast cancer families

**DOI:** 10.1186/s12885-019-5494-7

**Published:** 2019-04-04

**Authors:** Rajendra Bahadur Shahi, Sylvia De Brakeleer, Ben Caljon, Ingrid Pauwels, Maryse Bonduelle, Sofie Joris, Christel Fontaine, Marian Vanhoeij, Sonia Van Dooren, Erik Teugels, Jacques De Grève

**Affiliations:** 1 Laboratory of Medical and Molecular Oncology (LMMO), Vrije Universiteit Brussel (VUB), Brussels, Belgium; 2 Brussels Interuniversity Genomics High Throughput core (BRIGHTcore) platform, Universitair Ziekenhuis Brussel (UZ Brussel) / Vrije Universiteit Brussel (VUB), Brussels, Belgium; 3 Breast Cancer Clinic, Oncologisch Centrum, Universitair Ziekenhuis Brussel (UZ Brussel), Brussels, Belgium; 4 Familial Cancer Clinic, Oncologisch Centrum, Universitair Ziekenhuis Brussel (UZ Brussel), Brussels, Belgium; 5 Centre for Medical Genetics, Reproduction and Genetics, Universitair Ziekenhuis Brussel (UZ Brussel) / Vrije Universiteit Brussel (VUB), Brussels, Belgium

**Keywords:** Familial breast cancer, Missing heritability, *BRCA1* and *BRCA2*-negative, Whole exome sequencing, Candidate breast cancer predisposing genes/variants

## Abstract

**Background:**

In the majority of familial breast cancer (BC) families, the etiology of the disease remains unresolved. To identify missing BC heritability resulting from relatively rare variants (minor allele frequency ≤ 1%), we have performed whole exome sequencing followed by variant analysis in a virtual panel of 492 cancer-associated genes on BC patients from *BRCA1* and *BRCA2* negative families with elevated BC risk.

**Methods:**

BC patients from 54 *BRCA1* and *BRCA2*-negative families with elevated BC risk and 120 matched controls were considered for germline DNA whole exome sequencing. Rare variants identified in the exome and in a virtual panel of cancer-associated genes [492 genes associated with different types of (hereditary) cancer] were compared between BC patients and controls. Nonsense, frame-shift indels and splice-site variants (strong protein-damaging variants, called PDAVs later on) observed in BC patients within the genes of the panel, which we estimated to possess the highest probability to predispose to BC, were further validated using an alternative sequencing procedure.

**Results:**

Exome- and cancer-associated gene panel-wide variant analysis show that there is no significant difference in the average number of rare variants found in BC patients compared to controls. However, the genes in the cancer-associated gene panel with nonsense variants were more than two-fold over-represented in women with BC and commonly involved in the DNA double-strand break repair process. Approximately 44% (24 of 54) of BC patients harbored 31 PDAVs, of which 11 were novel. These variants were found in genes associated with known or suspected BC predisposition (*PALB2, BARD1, CHEK2, RAD51C* and *FANCA*) or in predisposing genes linked to other cancer types but not well-studied in the context of familial BC (*EXO1, RECQL4, CCNH, MUS81, TDP1, DCLRE1A, DCLRE1C, PDE11A* and *RINT1*) and genes associated with different hereditary syndromes but not yet clearly associated with familial cancer syndromes (*ABCC11, BBS10, CD96, CYP1A1, DHCR7, DNAH11, ESCO2, FLT4, HPS6, MYH8, NME8* and *TTC8*). Exome-wide, only a few genes appeared to be enriched for PDAVs in the familial BC patients compared to controls.

**Conclusions:**

We have identified a series of novel candidate BC predisposition variants/genes. These variants/genes should be further investigated in larger cohorts/case-control studies. Other studies including co-segregation analyses in affected families, locus-specific loss of heterozygosity and functional studies should shed further light on their relevance for BC risk.

**Electronic supplementary material:**

The online version of this article (10.1186/s12885-019-5494-7) contains supplementary material, which is available to authorized users.

## Background

Breast cancer is the most common cancer and the leading cause of cancer deaths among women in the world [1]. About 10–20% of all BC patients occur in a familial context, with multiple family members affected across generations [2]. Familial BC susceptibility resulting from deleterious germline variations located on chromosome 17q21 was brought to light through linkage analysis for the first time in 1990 [3]. Since then, many highly penetrant rare variants (with a relative risk of above 10-fold) in *BRCA1* (OMIM 113705)*, BRCA2* (OMIM 600185), *TP53* (OMIM 191170), *PTEN* (OMIM 601728), *STK11* (OMIM 602216) and *CDH1* (OMIM 192090) to moderately penetrant rare variants (with a relative risk of 2 to 4-fold) in *CHEK2* (OMIM 604373)*, PALB2* (OMIM 610355) [4]*, BARD1* (OMIM 601593)*, ATM* (OMIM 607585)*, BRIP1* (OMIM 605882) have been reported. The exact penetrance associated to pathogenic variants in several of these genes is still under investigation. These genes were identified through linkage analysis, positional cloning and/or candidate gene sequencing [5–17]. Furthermore, with the advent of DNA microarray technology, many low penetrant common variants (with a relative risk often much less than twofold) were unraveled through genome-wide association studies [18]. More recently, thanks to dramatic advances in the speed and scale of next-generation sequencing (NGS) technologies combined with sophisticated computation algorithms and a sharp decrease in sequencing cost, a path for the discovery of additional candidate BC predisposing variants has been opened. To name a few, variants in *XRCC2* (OMIM 600375)*, FANCC* (OMIM 613899)*, BLM* (OMIM 604610) and *PPM1D* (OMIM 605100) have been more recently reported as candidate variants with a BC risk through NGS technologies [19–21]. The aggregate currently known variants with high, moderate and low penetrance in familial BC susceptibility genes only account for up to 25–50% of all the high-risk BC families. This missing heritability in the remaining 50–75% of BC families [16, 22] reflects both the complexity of the BC genetic architecture and the challenges in identifying remaining BC predisposing variants for delivering timely screening, preventive intervention, and precision treatment.

Several studies have revealed that variants in familial BC susceptibility genes like *BRCA1, BRCA2*, *TP53, PALB2, CDH1, PTEN* (OMIM 601728)*, PIK3CA* (OMIM 171834)*, STK11* (OMIM 602216), *RINT1* (OMIM 610089) and *NF1* (OMIM 613113) are not only associated with BC predisposition, but also with a number of other malignancies [7, 9, 23–29]. In the current study, we hypothesized that in *BRCA1* and *BRCA2*-negative families with elevated BC risk, the analysis of a large array of genes previously associated to (hereditary) cancer syndromes or cancer in general, could likely lead to the identification of additional candidate BC predisposing genes/variants. Thus, firstly, we identified all rare variants both exome-wide and cancer-associated gene panel-wide (492 genes) in 54 BC patients from *BRCA1* and *BRCA2*-negative families with elevated BC risk and compared their relative incidence in 120 geographically matched controls. Secondly, all nonsense, frame-shift indels and splice-site variants detected in BC patients within the 492 genes of the panel, which we estimated to possess the highest probability to predispose to BC, were validated on an independent sequencing platform (Roche Junior).

## Methods

### Sample selection

A total of 57 BC patients and 120 controls were considered for this study. Among the BC patients (Additional file 1), 54 were from unrelated *BRCA1* and *BRCA2*-negative families with elevated BC and/or ovarian cancer (OC) risk (i.e. families with two or more affected first-degree relatives) and with a median age at diagnosis of 51 years (range: 36–72). The remaining three BC patients were included as “blinded internal positive controls”, each harboring a known germline variant in *BRCA1* (NM_007300.3:c.5096G > A), *BARD1* (NM_000465.3:c.1921C > T) or *PALB2* (NM_024675.3:c.1571C > G). All geographically matched unrelated controls considered in this study (patients consulted at the same hospital), sequenced according to the same wet lab protocol for cardiac arrhythmias, were unselected for personal or familial history of cancer. The overview of the process of sample preparation, sequencing, analysis and variant validation workflow is presented in ‘Additional file 2’.

Patient recruitment and blood sampling were performed according to the ethical procedures approved by the institutional ethics committee of the UZ Brussel. Peripheral blood was collected after obtaining a written informed consent for a broad genomic analysis covering also incidental findings in genes predictive for other diseases. Genomic DNA was prepared using Chemagic Magnetic Separation Module I (Chemagen) according to the manufacturer’s recommendations.

### A virtual panel of cancer-associated genes

After identification of rare variants in whole exomes, we further choose to prioritize variants present in a panel of 492 genes possibly/likely associated with (hereditary) cancer [hereafter called cancer-associated gene panel (CAGP) (Table 1 and Additional file 3)]. These genes are pooled together from seven gene lists: the well-known cancer susceptibility genes reported by Rahman et al. [30], various BC gene panels reported by Easton et al. [17], genes from BROCA-Cancer Risk Panel (Version 6) [31], Fanconi Anaemia pathway genes reported by Kanchi et al. [32], human DNA repair genes reported by Wood et al. [33], human cancer predisposition genes (GeneRead DNAseq Targeted Panel V2) from Qiagen and genes from the familial cancer database (FaCD, retrieved on 17/02/2015) [34]. Out of the 492 genes from our CAGP, 177 (36%) genes are contributed by at least two gene lists and are mostly known to be cancer susceptibility genes. The remaining 315 (64%) genes are private to a single gene list, mostly from Wood et al. (114 genes) and FaCD (167 genes). Some of these latter genes are not yet clearly associated with (hereditary) cancers (Table 1).

**Table 1:** Genes incorporated in the CAGP

Number of genes in common [number of gene lists containing these genes]	Genes in CAGP	Seven different gene lists with the number of contributing genes (n = total number of genes in the list)						
		Easton et al. (*n* = 119)	Rahman et al. (*n* = 114)	BROCA v6 (*n* = 61)	GeneRead v2 (Qiagen) (*n* = 143)	Kanchi et al. (*n* = 47)	Wood et al. (*n* = 178)	FaCD (*n* = 319)
8 [7]	*ATM, BRCA1, BRCA2, BRIP1, CHEK2, MLH1, PALB2, RAD51C*	8	8	8	8	8	8	8
17 [6]	*BLM, ERCC4, ERCC5, FANCA, FANCC, FANCG, MSH2, MSH6, MUTYH, NBN, PMS2, POLD1, POLE, RAD51D, SLX4, TP53, XPC*	17	16	10	17	8	17	17
36 [5]	*APC, ATR, BAP1, BMPR1A, CDH1, CDK4, CDKN2A, DDB2, ERCC2, ERCC3, FANCB, FANCD2, FANCE, FANCF, FANCI, FANCL, FANCM, FH, FLCN, MEN1, MRE11A, NF1, PRKAR1A, PTCH1, PTEN, RB1, RECQL4, RET, SDHB, SDHC, SDHD, SMAD4, STK11, VHL, WRN, XPA*	36	27	23	36	8	15	35
36 [4]	*ALK, AXIN2, BARD1, BUB1B, CDC73, CHEK1, CYLD, DICER1, DIS3L2, EPCAM, EXT1, EXT2, GATA2, GPC3, HRAS, KIT, MAX, MET, NF2, PALLD, PIK3CA, POLH, PRSS1, RAD51B, RHBDF2, RUNX1, SBDS, SDHAF2, SMARCA4, SMARCB1, SUFU, TMEM127, TSC1, TSC2, WT1, XRCC2*	33	29	10	34	0	4	34
24 [3]	*AKT1, CDKN1B, CEBPA, CTNNA1, DKC1, EGFR, EXO1, FAH, GALNT12, GEN1, GREM1, HFE, HOXB13, MLH3, PDGFRA, PHOX2B, RAD50, RAD51, SDHA, TERT, TGFBR1, UROD, WAS, XRCC3*	12	13	6	15	2	6	18
56 [2]	*AIP, ANTXR1, ANTXR2, ATRIP, CBL, CD96, CDKN1C, CEP57, COL7A1, CTNNB1, EHBP1, ELANE, EME1, EPHB2, ERCC1, ERCC6, ESCO2, FAM175A, FAS, GBA, GJB2, GLI3, GLMN, HMBS, HNF1A, KDR, LIG4, LYST, MC1R, MITF, MTAP, NSD1, PDE11A, PMS1, POLI, POLK, PRF1, PTCH2, PTPN11, REV3L, RINT1, RMRP, RNASEL, RPA1, RPA2, RPA4, RSPO1, SERPINA1, SH2D1A, SOS1, SRY, STAT3, TINF2, TP53BP1, TRIM37, UNG*	10	16	2	21	9	14	40
315 [1]	*ABCB11, ABCC11, ACVRL1, ADA, ADH1C, AFP, AICDA, AIRE, AKR1A1, ALKBH2, ALKBH3, AMH, AP3B1, APEX1, APEX2, APITD1, APLF, APTX, AR, ARL6, ASCC1, ATP2C1, ATP7B, ATP8B1, AXIN1, BBS1, BBS10, BBS12, BBS2, BBS4, BBS5, BBS7, BBS9, BLOC1S3, BRAF, BTK, CCM2, CCNH, CD40, CD40LG, CDK7, CDKN1A, CDKN2B, CDKN2C, CDKN2D, CETN2, CFTR, CHAF1A, CLK2, COL17A1, COL4A5, COL4A6, CREBBP, CSH1, CXCR4, CYP11B1, CYP11B2, CYP1A1, CYP21A2, CYP2E1, CYP7A1, DAPK1, DCLRE1A, DCLRE1B, DCLRE1C, DDB1, DHCR7, DMC1, DNAAF1, DNAAF2, DNAH11, DNAH5, DNAI1, DNAI2, DOCK8, DTNBP1, DUT, ELAC2, EME2, ENDOV, ENG, EP300, EPAS1, ERCC8, EZH2, F12, FAAP20, FAAP24, FAN1, FEN1, FGFR2, FGFR3, FHIT, FLG, FLT4, FMR1, FOXC2, FOXI1, FSHR, G6PC, GALT, GBE1, GNAS, GRB10, GSTM1, GSTT1, GTF2H1, GTF2H2, GTF2H3, GTF2H4, GTF2H5, H19, H2AFX, HAX1, HBB, HELQ, HES1, HLA-A, HLA-B, HLA-DQB1, HLA-DRA, HLA-DRB1, HLTF, HMGA2, HNF1B, HPS1, HPS3, HPS4, HPS5, HPS6, HUS1, IDH1, IDH2, IL1RN, IL2RG, ITK, JAG1, JAK2, KCNQ1OT1, KIF1B, KITLG, KLHDC8B, KRAS, KRIT1, KRT17, LHCGR, LIG1, LIG3, LMNA, LMX1B, LZTR1, MAD2L2, MAP2K1, MAP2K2, MBD4, MDC1, MGMT, MKKS, MMS19, MNAT1, MNX1, MPG, MPLKIP, MSH3, MSH4, MSH5, MSMB, MSR1, MUS81, MYH8, MYH9, NABP2, NAT2, NDN, NDUFA13, NEIL1, NEIL2, NEIL3, NHEJ1, NME8, NOTCH2, NRAS, NTHL1, NTRK1, NUDT1, OCA2, OGG1, PARP1, PARP2, PARP3, PAX5, PAX6, PCA3, PCNA, PDCD10, PDK1, PDK2, PDPK1, PER1, PMS2CL, PMS2P3, PNKP, POLB, POLG, POLL, POLM, POLN, POLQ, POT1, POU6F2, PPM1D, PPOX, PRKDC, PRPF19, PTH1R, PTPRJ, RAD1, RAD17, RAD18, RAD23A, RAD23B, RAD52, RAD54B, RAD54L, RAD9A, RAF1, RAG1, RAG2, RBBP8, RDM1, RECQL, RECQL5, REV1, RGS17, RIF1, RMI1, RMI2, RNF139, RNF168, RNF4, RNF8, RPA3, RPS19, RPS20, RRM2B, RSPH4A, RSPH9, RTEL1, SART3, SERPING1, SETBP1, SETMAR, SFTPA2, SH3BP2, SHFM1, SHOX, SHPRH, SIL1, SLC25A13, SLC26A4, SLC37A4, SLX1A, SLX1B, SMARCAL1, SMARCE1, SMC1A, SMC3, SMUG1, SNRPN, SPINK1, SPO11, SPRED1, SPRTN, SPRY4, SQSTM1, STS, STX11, T, TDG, TDP1, TDP2, TELO2, TERC, TGFBR2, TMC6, TMC8, TNF, TNFRSF11A, TNFRSF13B, TOP3A, TOP3B, TOPBP1, TP63, TREX1, TREX2, TRIM32, TRPS1, TTC8, TWIST1, TYR, TYRP1, UBE2A, UBE2B, UBE2N, UBE2V2, UNC13D, USP1, UVSSA, WDR48, XAB2, XIAP, XRCC1, XRCC4, XRCC5, XRCC6*	3	5	2	12	12	114	167

This panel of genes is the aggregation of seven different lists of genes found in the literature. The name and number of genes contributed by each gene list is indicated

### Target-enrichment and next-generation sequencing

For each of the BC patients and controls, one μg of DNA was fragmented using adaptive focused acoustics (Covaris) in order to obtain fragments of approximately 250 base pairs. After DNA end repair and adenylation, oligonucleotides adapters for paired-end sequencing (Illumina) were ligated to both ends of the fragments. Two hundred nanogram of ligated DNA of selected size was PCR amplified and subsequently captured by hybridization for 65 h with the Roche SeqCap EZ Human Exome v3.0 (Roche) Capture Library. After further selection of the targeted fragments through multiple steps of washing, the captured probe-selected DNA was cluster amplified on the Illumina cBot according to manufacturer’s protocol (Illumina), using five samples per flow cell lane in order to get sufficient DNA for the subsequent sequencing run. Sequencing was performed on a HiSeq1500 (Illumina) with a paired-end module, generating 125 base reads.

### Sequence alignment, variant calling and annotation

Primary processing including base calling, read filtering and adapter trimming were performed using the standard Illumina pipeline. High quality reads for each sample were mapped to the human genome reference assembly GRh37/hg19 (https://www.ncbi.nlm.nih.gov/grc/human/issues/HG-37, build 37.2, Feb 2009) using BWA-MEM [35] (http://bio-bwa.sourceforge.net/, version 0.7.10-r789) with the default setting. After marking PCR duplicates with Picard (https://broadinstitute.github.io/picard/, version 1.97), the GATK pipeline [36] (https://software.broadinstitute.org/gatk/, version 3.4–46) with GATK Best Practices guideline was followed for local indel-realignment, base recalibration, variants calling (HaplotypeCaller), variant recalibration and variant filtration. The variants obtained thereafter were annotated with ANNOVAR [37] (http://annovar.openbioinformatics.org/, version 2015-12-14) to refGene database and population databases (1000g2015aug_eur,1000g2015aug_all, esp6500siv2_ea, esp6500siv2_all, exac03nontcga, snp132NonFlagged and GoNL [38]) in addition to ljb26_all, a database for variant function prediction scores. All the databases were obtained from ANNOVAR website except GoNL (http://www.nlgenome.nl/, release 5).

### Variant filtration and classification

In-house Python script was used for variant filtration in three steps. Firstly, variants were only retained if they passed VQSLOD (tranche sensitivity threshold of 99.9%) and are located in the exons or at the splice-sites (±2 bp from the exon-intron border). In addition, we required a 10X absolute read depth at the variant position, at least two reads harboring the variant and a variant allele ratio between 20 and 80% along with a minor allele frequency (MAF) ≤1% in any of the population databases (mentioned earlier). Further, we assumed that those variants present in > 10% both in BC patients and controls most likely resulted from sequencing or alignment errors or they should be common variants exclusively in our study population (and thus missed by the MAF restriction). Thus, these variants were removed. Furthermore, missense variants were classified as “probably damaging” (pph2-prob ≥0.957), “possibly damaging” (0.453 ≤ pph2-prob≤0.956), or “benign” (pp2_hdiv ≤0.452) according to PolyPhen-2 (HDIV) [39] in silico prediction scores. Secondly, exome-wide variants that passed all the filters in the first stage were selected for their presence in genes of the CAGP. Lastly, frame-shift indels, nonsense and splice-site variants (hereafter collectively called potentially Protein Damaging Allelic Variants (PDAVs) as they have the highest probability to cause loss of protein function and thus to be associated to BC predisposition) that are present in genes of the CAGP were further validated.

### Variant validation

For validation of the PDAVs obtained from the Illumina platform using capture-based library enrichment system, an orthogonal approach using amplicon-based library enrichment on a 454 platform from Roche (Junior) was performed. Primer pairs were designed in order to amplify DNA fragments (amplicons) that contain the desired variants. One primer of the primer pair was designed towards intronic regions, when possible, to avoid amplification of processed pseudogenes. In addition, BLAST of the target sequence was performed in order to choose only primer pairs that specifically amplify the target region meanwhile avoiding non-specific or pseudo-gene amplification. Furthermore, primers binding to target sequences containing SNPs with a MAF > 1% were avoided. For variant analysis, SeqNext software (JSI medical systems) was used.

## Results

### Exome coverage

On average, about 1.0 × 10^8^ unique good quality reads were generated per exome both for BC patients and controls. About 87% of these reads from BC patients (controls: 86%) could be aligned to the reference genome covering 94% (controls: 95%) [BC patients range: 77–96%, controls range: 90–96%] of the exome with at least 10X target bases coverage. The median of ‘mean depth coverage’ at target region was about 107X and 101X [BC patients range: 46X-295X, controls range: 64X-148X] across all the BC patients and controls, respectively (Additional file 4). Coverage in CAGP was very similar to the coverage in exome both for BC patients and controls.

### Exome- and CAGP-wide variant enrichment in BC patients versus controls

Exome-wide, a total of 3,316,630 variants (average: 61,419 variants/BC patient) were called in 54 BC patients (3 internal positive controls excluded) and 7,413,256 variants (average: 61,777 variants/control) were called in 120 controls. After exhaustive variant filtering (as described in methods), 22,724 variants (average: 421 variants/BC patient) were retained in BC patients. Among them, 8153 single nucleotide variants (SNVs) were synonymous, 432 were in-frame indels, 543 were frame-shift indels, 162 were splice-site SNVs, 303 were nonsense SNVs and 5182 + 2227 + 5722 were missenses SNVs (predicted as “probably damaging”, “possibly damaging” and “benign” by PolyPhen-2, respectively). Similarly, in the controls we retained 51,219 variants (average: 427 variants/control) after filtering consisting of 17,891 synonymous SNVs, 981 in-frame indels, 1052 frame-shift indels, 420 splice-site SNVs, 768 nonsense SNVs and 11,929 + 5197 + 12,981 missenses SNVs (predicted as “probably damaging”, “possibly damaging” or “benign”, by PolyPhen-2, respectively). An overview of these data is presented in ‘Additional file 5’.

Subsequently, we investigated whether an exome-wide enrichment can be observed in the number of variants when comparing BC patients to controls (Student’s *t*-test or Welch’s *t*-test). No significant difference was observed in the average number of variants between BC patients and controls either by pooling all the variant types together (BC patients: controls; 420.81: 426.83, *p* = 0.3071) or by separately analyzing each sub-type of variants [synonymous SNVs (150.98: 149.09, *p* = 0.5058), in-frame indels (8.00: 8.18, *p* = 0.7043), splice-site SNVs (3.00: 3.50, *p* = 0.1053) and the missense SNVs [“probably damaging” (95.96: 99.41, *p* = 0.0628), “possibly damaging” (41.24: 43.31, *p* = 0.0866) and “benign” (105.96: 108.18, *p* = 0.3215)], except for frame-shift indels (10.06: 8.77, *p* = 0.0199) and nonsense SNVs (5.61: 6.40, *p* = 0.0446), (Additional file 6).

In the next step, we only considered the variants present in the 492 cancer-associated genes from the CAGP panel (see methods). In the BC patients, after filtering, we retained 240 synonymous SNVs, 8 in-frame indels, 13 frame-shift indels, 6 splice-site SNVs, 14 nonsense SNVs and 195 + 94 + 215 missenses SNVs (predicted as “probably damaging”, “possibly damaging” and “benign”, respectively). In the controls we retained 589 synonymous SNVs, 21 in-frame indels, 23 frame-shift indels, 20 splice-site SNVs, 13 nonsense SNVs and 398 + 174 + 417 missense SNVs (see Additional file 5). When comparing the average number of variants in BC patients versus controls, we observed that the average number of nonsense SNVs was more than twice higher in BC patients [BC patients: controls; 0.26:0.11; ratio = 2.39; *p* = 0.0287 (0.0688 with Welch correction)], whereas no obvious enrichment could be observed in the other sub-types of variants (see Additional file 6).

To investigate further whether specific genes are more frequently mutated in our BC patients compared to controls, we selected exome wide all the genes harboring high impact mutations (PDAVs) in at least two BC patients (see Additional file 7). Among the 95 genes selected, five (*FAM11B, GRAMD2, SP100, USP45* and *ZNF534*) can be considered candidate BC predisposing genes as they were mutated in three BC patients (out of 54) but not in any of the 120 controls (Additional file 8). Two other good candidate genes are *ASPH* and *C17orf80* as they harbored PDAVs in respectively five and four BC patients and only one control sample (Additional file 8). All PDAVs found in these 7 candidate BC predisposing genes were visually verified using the Integrative Genomics Viewer (IGV) [40].

### Validation of PDAVs within the CAGP

PDAVs resulting in dramatic changes in protein structure and function have the highest chance to be associated with BC predisposition. Those PDAVs that were detected in BC patients, passed the filters, and are located within the genes of the CAGP, were further validated on an independent sequencing platform (see methods) and were also reviewed manually using IGV [40]. Thirty-one out of 33 PDAVs present in 24 out of 54 BC patients (~ 44%) passed the validation step (Table 2), of which 11 PDAVs are not reported in dbSNP147. Among the BC patients with a PDAV, eighteen harbored a PDAV in a single gene, five harbored PDAVs in two genes and one harbored a PDAV in three genes. Furthermore, all five splice-site SNVs (Additional file 9) were considered disruptive by the in silico web-based tool “Human Splicing Finder” [41] (http://www.umd.be/HSF3/, release 3.0). Three of the 26 genes harboring PDAVs in the BC patients were also found mutated in the control samples, suggesting that these genes (*ABCC1, BBS10* and *PDE11A*) are not involved in cancer predisposition (compare Additional files 10 and 11), In addition, the pathogenic variants present in the three internal positive control samples included in this study were also identified.

**Table 2:** List of genes with the corresponding PDAVs that were validated as true positive in the corresponding BC patient

Gene	Variant type	Transcript: Base change (Protein change)	Exon (Intron)	MAF 1000 g [gnomAD]	rsID (dbsnp147)	BC Patients	Controls
*ABCC11*	splice-site substitution	NM_032583.3:c.395 + 2 T > C (p.?)	4	− [0.000004061]	–	BB44	0
*ABCC11*	nonsense substitution	NM_032583.3:c.297G > A (p.Trp99*)	4	− [0.0007676]	rs145048685	BB12	1x
*BARD1*	nonsense substitution	NM_000465.3:c.1690C > T (p.Gln564*)	8	− [0.00002032]	rs587780021	BB13	0
*BBS10*	frameshift insertions	NM_024685.3:c.271dup (p.Cys91Leufs*5)	2	− [0.0005626]	rs549625604	BB15 **	1x
*BBS10*	frameshift insertions	NM_024685.3:c.1543_1546dup (p.Thr516Argfs*7)	2	− [−]	–	BB48	0
*CCNH*	frameshift deletion	NM_001239.3:c.643_646del (p.Thr215Profs*21)	5	− [0.000008149]	–	BB15**	0
*CD96*	frameshift insertions	NM_198196.2:c.766dup (p.Ile256Asnfs*13)	5	− [0.00001625]	rs766366613	BB10	0
*CD96*	nonsense substitution	NM_198196.2:c.1321C > T (p.Arg441*)	11	− [0.0001056]	rs201691670	BB54**	0
*CHEK2*	frameshift deletion	NM_001005735.1:c.1229del (p.Thr410Metfs*15)	12	0.001 [0.002077]	rs555607708	BB17	0
*CYP1A1*	frameshift deletion	NM_000499.3:c.1371del (p.Cys457*)	7	0.0006 [0.0009096]	rs561096394	BB1	0
*DCLRE1A*	nonsense substitution	NM_001271816.1:c.412C > T (p.Arg138*)	2	0.002 [0.00279]	rs41292634	BB41	0
*DCLRE1C*	nonsense substitution	NM_001033855.2:c.241C > T (p.Arg81*)	3	− [0.00001221]	rs121908156	BB33***	0
*DHCR7*	splice-site substitution	NM_001360.2::c.964-1G > C (p.?)	9(8)	0.0026 [0.003762]	rs138659167	BB21	0
*DNAH11*	frameshift deletion	NM_001277115.1:c.2081_2082del (p.Val694Glyfs*2)	12	− [−]	–	BB29**	0
*ESCO2*	frameshift deletion	NM_001017420.2:c.876_879del (p.Asp292Glufs*48)	4	− [0.00000409]	rs80359856	BB35**	0
*EXO1*	splice-site substitution	NM_006027.4:c.2212-1G > C (p.?)	13(12)	0.0012 [0.001644]	rs4150000	BB35**	0
*FANCA*	splice-site substitution	NM_000135.2:c.2152-2A > G (p.?)	24(23)	− [−]	–	BB45	0
*FLT4*	nonsense substitution	NM_182925.4:c.3048C > A (p.Cys1016*)	22	− [−]	–	BB38	0
*HPS6*	stop-loss substitution	NM_024747.5:c.2326 T > C (p.*776Argext*38)	1	− [0.0001577]	rs200206362	BB33***	0
*MUS81*	nonsense substitution	NM_025128.4:c.392G > A (p.Trp131*)	4	− [0]	–	BB7	0
*MYH8*	nonsense substitution	NM_002472.2:c.1209C > A (p.Cys403*)	13	0.0004 [0.001105]	rs144321381	BB31	0
*NME8*	splice-site substitution	NM_016616.4:c.454 + 1G > A (p.?)	8	− [0.00006108]	rs538425312	BB3**	0
*NME8*	nonsense substitution	NM_016616.4:c.1600C > T (p.Arg534*)	17	0.0008 [0.0003171]	rs142525551	BB33***	0
*PALB2*	frameshift insertions	NM_024675.3:c.1674dup (p.Gln559Serfs*19)	4	− [−]	–	BB36	0
*PDE11A*	frameshift deletion	NM_016953.3:c.1660del (p.Cys554Valfs*14)	9	0.0008 [0.001214]	rs573163079	BB5	1x
*RAD51C*	frameshift deletion	NM_058216.2:c.181_182del (p.Leu61Alafs*11)	2	− [0.00001624]	rs754525165	BB54**	0
*RECQL4*	frameshift deletion	NM_004260.3:c.1573del (p.Cys525Alafs*33)	9	− [0.0002387]	rs386833845	BB52	0
*RECQL4*	frameshift deletion	NM_004260.3:c.3439del (p.Leu1147Cysfs*3)	20	− [−]	–	BB34	0
*RINT1*	nonsense substitution	NM_021930.4:c.64G > T (p.Glu22*)	2	− [−]	–	BB3**	0
*TDP1*	frameshift deletion	NM_018319.3:c.502del (p.Leu168Serfs*45)	3	− [0.00002039]	rs762302264	BB32	0
*TTC8*	nonsense substitution	NM_001288781.1:c.736C > T (p.Gln246*)	9	− [−]	–	BB29**	0

In addition to the details of each variant (variant type, transcript ID, base change, protein change, exon/intron), its frequency in controls, dbsnp147, global MAF in 1000 genome [2015 August release] and gnomAD [Ensembl GRCh37 release 95] are also given. ** = BC patient with PDAV in two genes, *** = BC patient with PDAV in three genes and “−” = not available

## Discussion

**Fig. 1:**
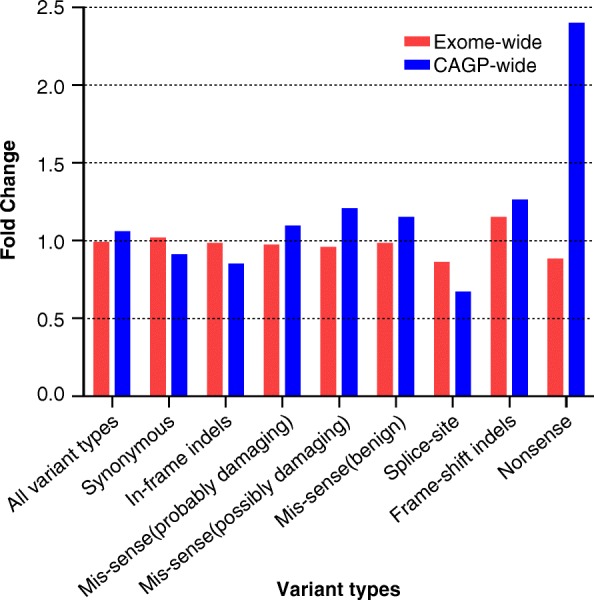
Fold change in the number of germline variants (by category) in BC patients compared to controls when considering the whole exome or the CAGP

It is expected that exome-wide NGS analysis of a germline DNA sample will reveal many variants when compared to a haploid reference genome, even when only rare variants (MAF ≤ 0.01) are taken into consideration. However, when comparing the total number of variants detected in two individuals of the same ethnicity we do not expect to find significant differences. We confirmed this assumption by (using the same wet bench and dry bench approaches) comparing the average number of variants found in persons belonging to two groups living in the same area (patients recruited in the same hospital): BC patients belonging to elevated risk BC families and controls not selected for personal or familial history of cancer but for cardiac arrhythmias. The ratio of average number of observed (rare) variants in both groups is very close to one for all types of variants (Fig. 1 (red) and Additional file 6) except for splice site and nonsense variants (0.86 and 0.88, respectively), probably because of the relatively small number of splice site and nonsense variants detected per BC patient and control. When focusing exclusively on the genes of the CAGP, similar observations were obtained (Fig. 1 (blue) and Additional file 6) except for the category of nonsense variants, where more than a two-fold excess of nonsense variants was detected in BC patients compared to controls (ratio = 2.39). Although a larger sample size is a minimal requirement to reach statistical significance, our data suggest that the nonsense variants found in excess in the genes of the CAGP among the BC patients (compared to controls) are implicated in the molecular mechanism modulating BC risk(about 50% of these nonsense variants). If the increased number of nonsense variants seen in BC patients is associated with increased cancer risk, one would expect that these nonsense variants will be more frequently identified in genes functionally correlated with the cancer predisposition process. To verify this assumption, the PANTHER over-representation Test (Released 20,171,205) [42] was used with a false discovery rate (FDR) < 0.05. This over-representation test compares a test gene list to a reference gene list and determines whether a particular class of genes (e.g. those associated to a specific biological process) is overrepresented or underrepresented. We found that genes involved in the DNA repair process, namely inter-strand cross-link repair (FDR: 4.94E-02), double-strand break (DSB) repair via nonhomologous end joining (FDR: 4.35E-02), non-recombinational repair (FDR: 4.42E-02), DSB repair (FDR: 8.35E-02) were overrepresented in BC patients while not in the controls. Indeed, four nonsense variants (out of 14) found in BC patients were found in genes involved in the DSB repair process while only one such variant (out of 13) was found among the controls. It remains unclear for us why the same phenomenon is not observed with the frameshift indels. It is possible that false positive indel calls masked a possible enrichment of the true positive frameshift indels.

Our exome wide analysis revealed only seven genes (*ASP, C17orf80, FAM111B, GRAMD2, SP100, USP45* and *ZNF534*) with high impact mutations (PDAVs) in three (or more) BC patients while comparable mutations were not found (or only once) among the control samples. None of these genes was reported to possess cancer predisposing properties and therefore not included in the CAGP. Gene Ontology (GO) annotation [43] for molecular function, biological processes and Reactome Pathways indicated that *SP100* and *USP45* are involved in DNA repair while *ZNF534* is involved in DNA-templated regulation of transcription, making them good candidate cancer predisposing genes. No molecular function or biological process was annotated to *C17orf80* and *FAM11B*, whereas *ASPH* and *GRAMD2* were reported to be involved in calcium homeostasis and transport (Additional file 8).

When restricting our variant analyses performed on BC patients to the 492 genes of the CAGP, we found novel as well as known PDAVs in several genes known or suspected to be BC predisposing (Table 2 and Additional file 10). Genes participating in DNA DSB repair process e.g. *PALB2*, *BARD1*, *CHEK2* and *RAD51C* are particularly intriguing as DSB repair process defective tumors can be selectively targeted by PARP (poly (ADP-ribose) polymerase) inhibitors resulting in synthetic lethality [44–46]. We also found PDAVs in genes linked to DNA repair, FA or occurring in some types of cancers but not well studied in the context of familial BC (Table 2 and Additional file 10). These candidate BC predisposing genes are also interesting to scrutinize further in familial BC setting as it is known that familial BC susceptibility genes can also predispose to multiple cancers [30]. Furthermore, we detected PDAVs in genes associated with other hereditary syndromes but not clearly related to cancer (Table 2 and Additional file 10). These genes are mostly derived from the FaCD panel, which is uncurated. PDAVs detected in the CAGP from control samples but not present in the BC patients are listed in Additional file 11.

Only about 44% of the BC patients were found to harbor a PDAV in one (and exceptionally in two or three) gene(s) of the CAGP in this study. Eleven out of 31 PDAVs detected were not reported in dbSNP147 and therefore considered novel. We should keep in mind that these PDAVs are not necessarily BC predisposing. Therefore, their cancer predisposing attributes should be further investigated in much larger cohort /case-control studies or by performing co-segregation analyses in positive families (if sufficient families are available). Although in this study we mainly focused on candidate PDAVs found in genes of the CAGP (which only accounts for 2% of the full exome), we must remain aware that missense variants in genes of the CAGP but also PDAVs and missense variants outside this gene panel may also predispose to BC. For instance, we identified 7 genes not represented in the CAGP in which PDAVs were over-represented in the BC patient cohort. Moreover, BC predisposition may not necessarily rely solely on the presence of one particular variant in the family but may result from combinatorial interactions between several variants. Indeed, it has been proposed that in the majority of BC families, BC predisposition could be polygenic in nature and the contribution of several variants located in genes associated to moderate or low risk could be responsible for the increased susceptibility to BC [47]. The mechanism how these different variants cooperate at the molecular level to create an increased BC risk is a matter of further investigation [48].

## Conclusions

On average, twice more nonsense variants were found in BC patients than in controls when analyzing the genes from the CAGP. Moreover, GO analysis (biological process) of the genes accumulating those nonsense variants indicated that genes involved in the DSB repair process were overrepresented in the BC patients but not in controls. Comparable observations were not made for the other variant types in the CAGP, nor when considering the whole exome. Taken together, our observations might indicate that a nonsense variant found in the CAGP of a BC patient has more than 50% chance to be associated with BC risk while similar conclusions cannot be drawn for “probably/possibly damaging” missense or frameshift mutations. Larger case-control studies should be performed to confirm these assumptions and validate our candidates. This preliminary study in 54 BC patients from *BRCA1* and *BRCA2*-negative BC families with elevated cancer risk identified candidate BC predisposing PDAVs (known as well as unknown) in 30 genes; *PALB2, BARD1, CHEK2, RAD51C, FANCA, RINT1, EXO1, RECQL4, CCNH, MUS81, TDP1, DCLRE1A, DCLRE1C, CD96, CYP1A1, DHCR7, DNAH11, ESCO2, FLT4, HPS6, MYH8, NME8*, *TTC8, ASPH, C17orf80, FAM111B,GRAMD2, ZNF534, SP100* and *USP45*. The seven last genes of this list were not connected to the cancer process so far. These novel candidate variants and their associated genes should be further investigated with other methods to confirm their role in BC predisposition.

## Additional files

Additional file 1: Breast cancer (BC) patients considered for the study, their age at diagnosis and the number of affected first-degree relatives in their families. (XLSX 11 kb)

Additional file 2: Sample preparation, sequencing, analysis and variant validation workflow. (TIF 2207 kb)

Additional file 3: A panel of cancer-associated genes with their corresponding genomic coordinates and the lists of genes from which they are extracted. (XLSX 47 kb)

Additional file 4: Sequencing coverage details for each BC patient and control. (XLSX 23 kb)

Additional file 5: Types and number of variants detected (exome- and CAGP-wide) before and after variant filtration in each BC patient and control. (XLSX 32 kb)

Additional file 6: BC patients vs. controls variant types enrichment in exome- and CAGP-wide. (XLSX 10 kb)

Additional file 7: List with all genes (exome-wide) presenting a PDAV in at least two BC patients and the number of control samples presenting a PDAV in the same gene. (XLSX 12 kb)

Additional file 8: List with genes presenting a PDAV in three (or more) BC patients but not (or only once) in the control samples. GO annotations are shown for molecular function, biological processes and Reactome Pathways. (XLSX 13 kb)

Additional file 9: Splice-site variants with their corresponding HSF and MaxEnt Scores from Human Splicing Finder 3.0. (XLSX 10 kb)

Additional file 10: List with PDAVs detected in 54 BC patients located in genes linked to (hereditary) cancer and/or hereditary diseases. Each variant/gene is briefly commented. (DOCX 63 kb)

Additional file 11: PDAVs found in the CAPG of the 120 control samples and their occurrence in the general population. (XLSX 12 kb)

## Abbreviations

BC: breast cancer
CAGP: cancer-associated gene panel
DSB: double-strand break
FA: Fanconi anemia
FaCD: familial cancer database
FDR: false discovery rate
GO: Gene ontology
GoNL: Genome of the Netherlands
IGV: Integrative Genomics Viewer
MAF: minor allele frequency
NGS: next-generation sequencing
PDAV: protein-damaging allelic variant
VQSLOD: variant quality score log-odds

## References

[1] Torre LA, Bray F, Siegel RL, Ferlay J, Lortet-Tieulent J, Jemal A (2015). Global cancer statistics, 2012. CA Cancer J Clin.

[2] Rahman N, Stratton MR (1998). The genetics of breast cancer susceptibility. Annu Rev Genet.

[3] Hall JM, Lee MK, Newman B, Morrow JE, Anderson LA, Huey B, King MC (1990). Linkage of early-onset familial breast cancer to chromosome 17q21. Science.

[4] Antoniou AC, Casadei S, Heikkinen T, Barrowdale D, Pylkas K, Roberts J, Lee A, Subramanian D, De Leeneer K, Fostira F (2014). Breast-cancer risk in families with mutations in PALB2. N Engl J Med.

[5] Miki Y, Swensen J, Shattuck-Eidens D, Futreal PA, Harshman K, Tavtigian S, Liu Q, Cochran C, Bennett LM, Ding W (1994). A strong candidate for the breast and ovarian cancer susceptibility gene BRCA1. Science.

[6] Wooster R, Neuhausen SL, Mangion J, Quirk Y, Ford D, Collins N, Nguyen K, Seal S, Tran T, Averill D (1994). Localization of a breast cancer susceptibility gene, BRCA2, to chromosome 13q12-13. Science.

[7] Malkin D, Li FP, Strong LC, Fraumeni JF, Nelson CE, Kim DH, Kassel J, Gryka MA, Bischoff FZ, Tainsky MA (1990). Germ line p53 mutations in a familial syndrome of breast cancer, sarcomas, and other neoplasms. Science.

[8] Saal LH, Gruvberger-Saal SK, Persson C, Lovgren K, Jumppanen M, Staaf J, Jonsson G, Pires MM, Maurer M, Holm K (2008). Recurrent gross mutations of the PTEN tumor suppressor gene in breast cancers with deficient DSB repair. Nat Genet.

[9] Hearle N, Schumacher V, Menko FH, Olschwang S, Boardman LA, Gille JJ, Keller JJ, Westerman AM, Scott RJ, Lim W (2006). Frequency and spectrum of cancers in the Peutz-Jeghers syndrome. Clinical cancer research : an official journal of the American Association for Cancer Research.

[10] Masciari S, Larsson N, Senz J, Boyd N, Kaurah P, Kandel MJ, Harris LN, Pinheiro HC, Troussard A, Miron P (2007). Germline E-cadherin mutations in familial lobular breast cancer. J Med Genet.

[11] Meijers-Heijboer H, van den Ouweland A, Klijn J, Wasielewski M, de Snoo A, Oldenburg R, Hollestelle A, Houben M, Crepin E, van Veghel-Plandsoen M (2002). Low-penetrance susceptibility to breast cancer due to CHEK2(*)1100delC in noncarriers of BRCA1 or BRCA2 mutations. Nat Genet.

[12] Rahman N, Seal S, Thompson D, Kelly P, Renwick A, Elliott A, Reid S, Spanova K, Barfoot R, Chagtai T (2007). PALB2, which encodes a BRCA2-interacting protein, is a breast cancer susceptibility gene. Nat Genet.

[13] De Brakeleer S, De Greve J, Loris R, Janin N, Lissens W, Sermijn E, Teugels E (2010). Cancer predisposing missense and protein truncating BARD1 mutations in non-BRCA1 or BRCA2 breast cancer families. Hum Mutat.

[14] Broeks A, Urbanus JH, Floore AN, Dahler EC, Klijn JG, Rutgers EJ, Devilee P, Russell NS, van Leeuwen FE, van 't Veer LJ (2000). ATM-heterozygous germline mutations contribute to breast cancer-susceptibility. Am J Hum Genet.

[15] Seal S, Thompson D, Renwick A, Elliott A, Kelly P, Barfoot R, Chagtai T, Jayatilake H, Ahmed M, Spanova K (2006). Truncating mutations in the Fanconi anemia J gene BRIP1 are low-penetrance breast cancer susceptibility alleles. Nat Genet.

[16] Stratton MR, Rahman N (2008). The emerging landscape of breast cancer susceptibility. Nat Genet.

[17] Easton DF, Pharoah PD, Antoniou AC, Tischkowitz M, Tavtigian SV, Nathanson KL, Devilee P, Meindl A, Couch FJ, Southey M (2015). Gene-panel sequencing and the prediction of breast-cancer risk. N Engl J Med.

[18] Easton DF, Pooley KA, Dunning AM, Pharoah PD, Thompson D, Ballinger DG, Struewing JP, Morrison J, Field H, Luben R (2007). Genome-wide association study identifies novel breast cancer susceptibility loci. Nature.

[19] Hilbers FS, Wijnen JT, Hoogerbrugge N, Oosterwijk JC, Collee MJ, Peterlongo P, Radice P, Manoukian S, Feroce I, Capra F (2012). Rare variants in XRCC2 as breast cancer susceptibility alleles. J Med Genet.

[20] Thompson ER, Doyle MA, Ryland GL, Rowley SM, Choong DY, Tothill RW, Thorne H, kConFab BDR, Li J (2012). Exome sequencing identifies rare deleterious mutations in DNA repair genes FANCC and BLM as potential breast cancer susceptibility alleles. PLoS Genet.

[21] Ruark E, Snape K, Humburg P, Loveday C, Bajrami I, Brough R, Rodrigues DN, Renwick A, Seal S, Ramsay E (2013). Mosaic PPM1D mutations are associated with predisposition to breast and ovarian cancer. Nature.

[22] Melchor L, Benitez J (2013). The complex genetic landscape of familial breast cancer. Hum Genet.

[23] Mai PL, Chatterjee N, Hartge P, Tucker M, Brody L, Struewing JP, Wacholder S (2009). Potential excess mortality in BRCA1/2 mutation carriers beyond breast, ovarian, prostate, and pancreatic cancers, and melanoma. PLoS One.

[24] Jones S, Hruban RH, Kamiyama M, Borges M, Zhang X, Parsons DW, Lin JC, Palmisano E, Brune K, Jaffee EM (2009). Exomic sequencing identifies PALB2 as a pancreatic cancer susceptibility gene. Science.

[25] Norton JA, Ham CM, Van Dam J, Jeffrey RB, Longacre TA, Huntsman DG, Chun N, Kurian AW, Ford JM (2007). CDH1 truncating mutations in the E-cadherin gene: an indication for total gastrectomy to treat hereditary diffuse gastric cancer. Ann Surg.

[26] Figer A, Kaplan A, Frydman M, Lev D, Paswell J, Papa MZ, Goldman B, Friedman E (2002). Germline mutations in the PTEN gene in Israeli patients with Bannayan-Riley-Ruvalcaba syndrome and women with familial breast cancer. Clin Genet.

[27] Orloff MS, He X, Peterson C, Chen F, Chen JL, Mester JL, Eng C (2013). Germline PIK3CA and AKT1 mutations in Cowden and Cowden-like syndromes. Am J Hum Genet.

[28] Park DJ, Tao K, Le Calvez-Kelm F, Nguyen-Dumont T, Robinot N, Hammet F, Odefrey F, Tsimiklis H, Teo ZL, Thingholm LB (2014). Rare mutations in RINT1 predispose carriers to breast and lynch syndrome-spectrum cancers. Cancer discovery.

[29] Friedman JM: Neurofibromatosis 1. In: GeneReviews(R)*.* Edn. Edited by Pagon RA, Adam MP, Ardinger HH, Wallace SE, Amemiya A, Bean LJH, Bird TD, Fong CT, Mefford HC, Smith RJH *et al*. Seattle (WA); 1993.

[30] Rahman N (2014). Realizing the promise of cancer predisposition genes. Nature.

[31] Walsh T, Casadei S, Lee MK, Pennil CC, Nord AS, Thornton AM, Roeb W, Agnew KJ, Stray SM, Wickramanayake A (2011). Mutations in 12 genes for inherited ovarian, fallopian tube, and peritoneal carcinoma identified by massively parallel sequencing. Proc Natl Acad Sci U S A.

[32] Kanchi KL, Johnson KJ, Lu C, McLellan MD, Leiserson MD, Wendl MC, Zhang Q, Koboldt DC, Xie M, Kandoth C (2014). Integrated analysis of germline and somatic variants in ovarian cancer. Nat Commun.

[33] Wood Laboratory: Human DNA repair genes(last modified on Tuesday 15th April 2014**)**. http://scienceparkmdandersonorg/labs/wood/dna_repair_geneshtml, Accessed 7 Apr 2016.

[34] Sijmons RH: Identifying Patients with Familial Cancer Syndromes. In: *Cancer Syndromes.* edn. Edited by Riegert-Johnson DL, Boardman LA, Hefferon T, Roberts M. Bethesda (MD); 2009. 21249759

[35] Li H (2014). Toward better understanding of artifacts in variant calling from high-coverage samples. Bioinformatics.

[36] DePristo MA, Banks E, Poplin R, Garimella KV, Maguire JR, Hartl C, Philippakis AA, del Angel G, Rivas MA, Hanna M (2011). A framework for variation discovery and genotyping using next-generation DNA sequencing data. Nat Genet.

[37] Wang K, Li M, Hakonarson H (2010). ANNOVAR: functional annotation of genetic variants from high-throughput sequencing data. Nucleic Acids Res.

[38] Genome of the Netherlands C (2014). Whole-genome sequence variation, population structure and demographic history of the Dutch population. Nat Genet.

[39] Adzhubei IA, Schmidt S, Peshkin L, Ramensky VE, Gerasimova A, Bork P, Kondrashov AS, Sunyaev SR (2010). A method and server for predicting damaging missense mutations. Nat Methods.

[40] Robinson JT, Thorvaldsdottir H, Winckler W, Guttman M, Lander ES, Getz G, Mesirov JP (2011). Integrative genomics viewer. Nat Biotechnol.

[41] Desmet FO, Hamroun D, Lalande M, Collod-Beroud G, Claustres M, Beroud C (2009). Human splicing finder: an online bioinformatics tool to predict splicing signals. Nucleic Acids Res.

[42] Mi H, Muruganujan A, Casagrande JT, Thomas PD (2013). Large-scale gene function analysis with the PANTHER classification system. Nat Protoc.

[43] Ashburner M, Ball CA, Blake JA, Botstein D, Butler H, Cherry JM, Davis AP, Dolinski K, Dwight SS, Eppig JT (2000). Gene ontology: tool for the unification of biology. The gene ontology consortium. Nat Genet.

[44] Fong PC, Boss DS, Yap TA, Tutt A, Wu P, Mergui-Roelvink M, Mortimer P, Swaisland H, Lau A, O'Connor MJ (2009). Inhibition of poly(ADP-ribose) polymerase in tumors from BRCA mutation carriers. N Engl J Med.

[45] Lord CJ, Ashworth A (2016). BRCAness revisited. Nat Rev Cancer.

[46] Greve JD, Decoster L, Shahi RB, Fontaine C, Vanacker L, Pauwels I, Denayer E, Brakeleer SD, Teugels E (2016). Parp inhibitors. Belg J Med Onco.

[47] Antoniou AC, Easton DF (2006). Models of genetic susceptibility to breast cancer. Oncogene.

[48] Teugels E, De Brakeleer S (2017). An alternative model for (breast) cancer predisposition. NPJ Breast Cancer.

