# Supplementary figures and images for: Identification of candidate cancer predisposing variants by performing whole-exome sequencing on index patients from BRCA1 and BRCA2-negative breast cancer families
Source: BMC Cancer. 2019 Apr 4;19:313. doi: 10.1186/s12885-019-5494-7 (PMC6449945; doi:10.1186/s12885-019-5494-7)

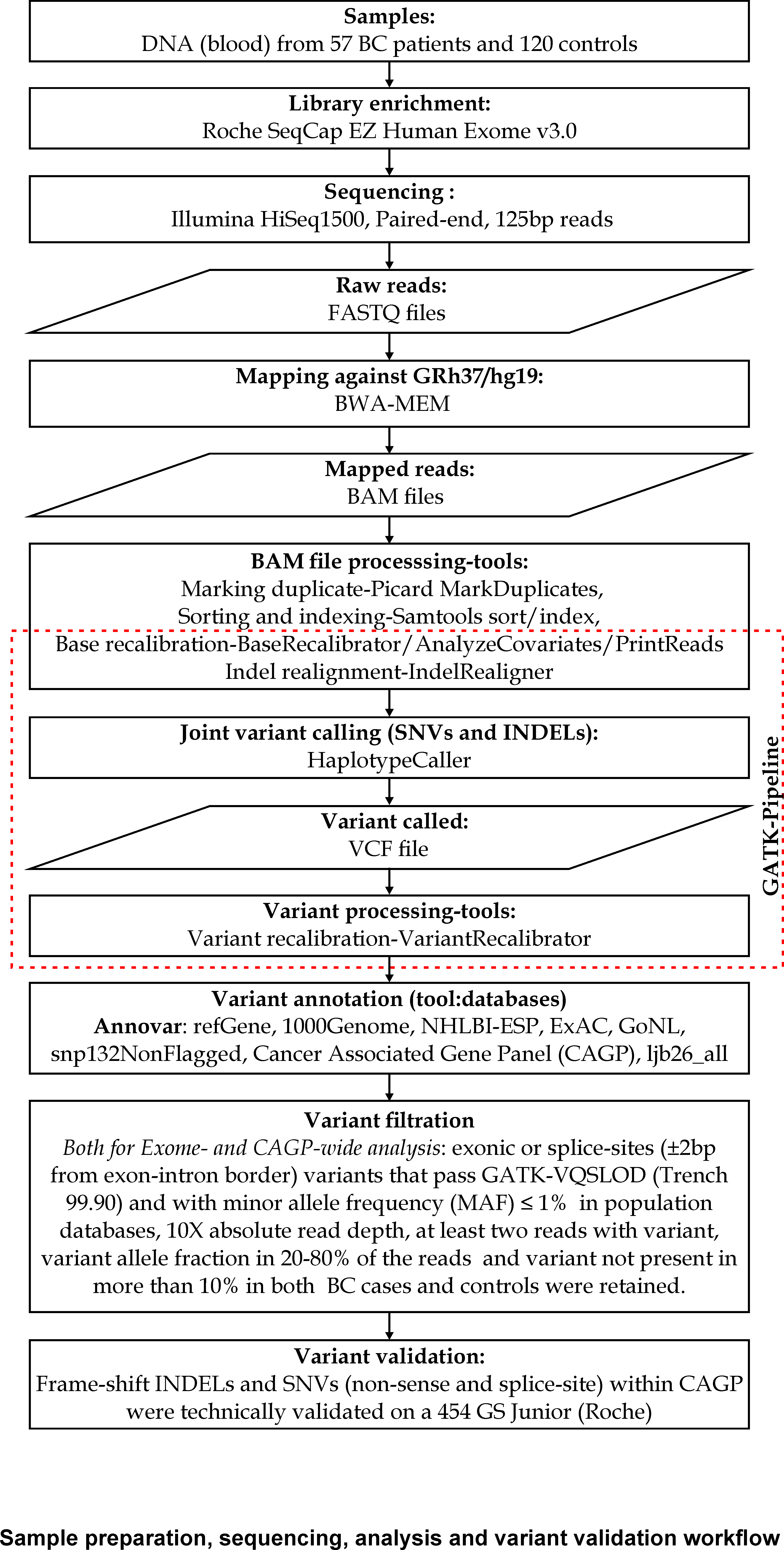

Supplement: Supplementary file 2 — Sample preparation, sequencing, analysis and variant validation workflow. (TIF 2207 kb) [file 12885_2019_5494_MOESM2_ESM.tif]
