# Supplementary material for: Identification of candidate cancer predisposing variants by performing whole-exome sequencing on index patients from BRCA1 and BRCA2-negative breast cancer families
Source: BMC Cancer. 2019 Apr 4;19:313. doi: 10.1186/s12885-019-5494-7 (PMC6449945; doi:10.1186/s12885-019-5494-7)
Supplement: Supplementary file 10 — List with PDAVs detected in 54 BC patients located in genes linked to (hereditary) cancer and/or hereditary diseases. Each variant/gene is briefly commented. (DOCX 63 kb) [file 12885_2019_5494_MOESM10_ESM.docx]

**Additional file 10:** List with PDAVs detected in 54 BC patients located in genes linked to (hereditary) cancer and/or hereditary diseases. Each variant/gene is shortly commented.

| Gene | Variant | Variant novelty | Description |
| --- | --- | --- | --- |
| **A. PDAVs in genes reported to be associated with BC or other cancer types** | | | |
| BARD1 | c.1690C>T(p.Gln564*) | Known | The variant was previously reported in a triple negative BC patient cohort by our group [[1](#_ENREF_1)]. Variants in BARD1 are not only reported in high-risk familial BC/OC but are also significantly enriched in familial neuroblastoma [[2](#_ENREF_2), [3](#_ENREF_3)] |
| CHEK2 | c.1229del(p.Thr410Metfs*15) | Known | The variant was observed in the kinase domain of CHEK2. This likely deleterious PDAV is located downstream of a c.1100del variant which is well-known to be associated with a two to five-fold increase in BC risk (depending on the familial context) [[4](#_ENREF_4)]. Recently, c.1229del was reported in cervical tumors but its germline nature was not confirmed in that study [[5](#_ENREF_5)] |
| FANCA | c.2152-2A>G(p.?) | Novel | FANCA (OMIM 607139) is involved in cell cycle checkpoint & DNA repair and variants in this gene are reported in FA and BC [[6](#_ENREF_6)] |
| PALB2 | c.1674dup(p.Gln559Serfs*19) | Novel | The variant was observed in the low complexity region of PALB2. Bi-allelic variants in PALB2 are reported to be associated with Fanconi anemia (FA), whereas mono-allelic variants are associated with an increased risk of hereditary BC and pancreatic cancers [[7-9](#_ENREF_7)] |
| RAD51C | c.181_182del(p.Leu61Alafs*11) | Known | Mono-allelic and bi-allelic variants in RAD51C are associated with an increased risk of BC/OC and FA-like disorder, respectively [[10](#_ENREF_10), [11](#_ENREF_11)] |
| **B. PDVs in genes linked to DNA repair, FA or occurring in some types of cancers but not well studied in the context of familial BC** | | | |
| RINT1 | c.64G>T(p.Glu22*) | Novel | RINT1 interacts with RAD50 and RBL2 (OMIM 180203) and participate in cell cycle checkpoints control after DNA damage as well as blocking of telomerase-independent telomere lengthening [[16](#_ENREF_16)]. Earlier, it was reported that variants in RINT1 are associated with breast and Lynch syndrome-spectrum cancers [[14](#_ENREF_14)]. However, a recent study in 2,024 familial BCs did not provide any support for RINT1 being a moderate-penetrant familial BC susceptibility gene. This larger study rather excluded a moderate effect of RINT1 in BC predisposition [[15](#_ENREF_15)]. |
| CCNH | c.643_646del(p.Thr215Profs*21) | Novel | Variants in CCNH (OMIM 601953)are reported to influence thyroid cancer susceptibility [[16](#_ENREF_16)] |
| DCLRE1A | c.412C>T(p.Arg138*) | Known | These genes are involved in the DSB repair process. DCLRE1A (OMIM 609682) is reported mutated in a northern Finnish familial BC patient whereas DCLRE1C (OMIM 605988) is reported mutated in a BRCA1/2 and PALB2 negative BC patient [[17](#_ENREF_17), [18](#_ENREF_18)]. |
| DCLRE1C | c.241C>T(p.Arg81*) | Known |  |
| EXO1 | c.2212-1G>C(p.?) | Known | EXO1, through its 5' to 3' exonuclease activity, is involved in mismatch repair and recombination after interacting with MSH2 (OMIM 609309). There are conflicting reports about EXO1 being associated with hereditary non-polyposis colorectal cancer [[19](#_ENREF_19)]. Recently, a truncating mutation in EXO1 was also reported in a Russian high-risk BC patient [[20](#_ENREF_20)] |
| MUS81 | c.392G>A(p.Trp131*) | Novel | This gene is involved in the DSB repair process. Dysregulated MUS81 (OMIM 606591) is linked to neurological diseases and cancer predisposition [[21](#_ENREF_21)] |
| PDE11A^+^ | c.1660del(p.Cys554Valfs*14) | Known | PDE11A (OMIM 604961) is involved in DNA repair pathways. It regulates signal transduction by controlling intracellular concentration of cAMP and cGMP. Germline variants in this gene are reported in Cushing syndrome, prostate cancer and testicular germ cell tumors [[22-24](#_ENREF_22)] |
| RECQL4 | c.1573del(p.Cys525Alafs*33) | Known | RECQL4 (OMIM 603780) is an essential helicase gene involved in DNA replication and repair. Rothmund-Thomson syndrome patients with RECQL4 variants have an increased risk for osteosarcoma [[25](#_ENREF_25)] |
|  | c.3439del(p.Leu1147Cysfs*3) | Novel |  |
| **C. PDAVs in genes associated with other hereditary syndromes but only remotely related to cancer** | | | |
| ABCC11^+^ | c.395+2T>C(p.?) | Novel | ABCC11 (OMIM 607040) responsible for transposrting various molecules across extra- and intra-cellular membranes.Although some reports associate variants in ABCC11 with wet-type earwax, axillary osmidrosis, colostrum secretion from the mammary gland, and potential BC susceptibility, these data are presently inconclusive [[26](#_ENREF_26)] |
|  | c.297G>A(p.Trp99*) | Known |  |
| BBS10^+^ | c.271dup(p.Cys91Leufs*5) | Known | Both BBS10 (OMIM 610148) and TTC8 (OMIM 608132) are associated with the Bardet-Biedl syndrome. In addition to other anomalies, they are also involved in poor breast bud development [[27](#_ENREF_27)] |
|  | c.1543_1546dup(p.Thr516Argfs*7) | Novel |  |
| CYP1A1 | c.1371del(p.Cys457*) | Known | CYP1A1 (OMIM 108330) is involved in drug, cholesterol, steroids and other lipid metabolism and is associated with lung and colorectal cancer [[28](#_ENREF_28)] |
| ESCO2 | c.876_879del(p.Asp292Glufs*48) | Known | Dysregulation of cohesion and cohesin-associated genes (like ESCO2 (OMIM 609353) are linked to cancers like esophageal adenocarcinoma, melanoma, rhabdomyosarcoma and oculomotor nerve angioma [[29](#_ENREF_29)] |
| TDP1 | c.502del(p.Leu168Serfs*45) | Known | TDP1 (OMIM 607198) is essential for repairing stalled topoisomerase I-DNA complexes during DNA replication and transcription. Variants in TDP1 are reported in Spine Cerebellar Ataxia with Axonal Neuropathy [[30](#_ENREF_30)] |
| CD96 | c.766dup(p.Ile256Asnfs*13) | Known | PDAVs were also found in CD96 (OMIM 606037), (OMIM 602858), DNAH11 (OMIM 603339), FLT4 (OMIM 136352), HPS6 (OMIM 607522), MYH8 (OMIM 160741), NME8 (OMIM 607421), while variants in these genes have been found associated with C syndrome, Smith-Lemli-Opitz Syndrome, Primary ciliary dyskinesia, Hereditary lymphedema, Hermansky-Pudlak Syndrome 6, Trismus-Pseudocamptodactyly Syndrome, and Primary ciliary dyskinesia, respectively. Two of these genes were found mutated more than once in BC patients but not mutated in controls. |
|  | c.1321C>T(p.Arg441*) | Known |  |
| DHCR7 | c.964-1G>C(p.?) | Known |  |
| DNAH11 | c.2081_2082del(p.Val694Glyfs*2) | Novel |  |
| FLT4 | c.3048C>A(p.Cys1016*) | Novel |  |
| HPS6 | c.2326T>C(p.*776Argext*38) | Known |  |
| MYH8 | c.1209C>A(p.Cys403*) | Known |  |
| NME8 | c.454+1G>A(p.?) | Known |  |
|  | c.1600C>T(p.Arg534*) | Known |  |
| TTC8 | c.736C>T(p.Gln246*) | Novel |  |

**^+^** gene presenting PDAVs in controls

1. De Brakeleer S, De Greve J, Desmedt C, Joris S, Sotiriou C, Piccart M, Pauwels I, Teugels E: **Frequent incidence of BARD1-truncating mutations in germline DNA from triple-negative breast cancer patients**. *Clin Genet* 2016, **89**(3):336-340.

2. Karppinen SM, Heikkinen K, Rapakko K, Winqvist R: **Mutation screening of the BARD1 gene: evidence for involvement of the Cys557Ser allele in hereditary susceptibility to breast cancer**. *Journal of medical genetics* 2004, **41**(9):e114.

3. Pugh TJ, Morozova O, Attiyeh EF, Asgharzadeh S, Wei JS, Auclair D, Carter SL, Cibulskis K, Hanna M, Kiezun A *et al*: **The genetic landscape of high-risk neuroblastoma**. *Nature genetics* 2013, **45**(3):279-284.

4. Weischer M, Bojesen SE, Ellervik C, Tybjaerg-Hansen A, Nordestgaard BG: **CHEK2*1100delC genotyping for clinical assessment of breast cancer risk: meta-analyses of 26,000 patient cases and 27,000 controls**. *Journal of clinical oncology : official journal of the American Society of Clinical Oncology* 2008, **26**(4):542-548.

5. Muller E, Brault B, Holmes A, Legros A, Jeannot E, Campitelli M, Rousselin A, Goardon N, Frebourg T, Krieger S *et al*: **Genetic profiles of cervical tumors by high-throughput sequencing for personalized medical care**. *Cancer medicine* 2015, **4**(10):1484-1493.

6. Seal S, Barfoot R, Jayatilake H, Smith P, Renwick A, Bascombe L, McGuffog L, Evans DG, Eccles D, Easton DF *et al*: **Evaluation of Fanconi Anemia genes in familial breast cancer predisposition**. *Cancer research* 2003, **63**(24):8596-8599.

7. Rahman N, Seal S, Thompson D, Kelly P, Renwick A, Elliott A, Reid S, Spanova K, Barfoot R, Chagtai T *et al*: **PALB2, which encodes a BRCA2-interacting protein, is a breast cancer susceptibility gene**. *Nature genetics* 2007, **39**(2):165-167.

8. Reid S, Schindler D, Hanenberg H, Barker K, Hanks S, Kalb R, Neveling K, Kelly P, Seal S, Freund M *et al*: **Biallelic mutations in PALB2 cause Fanconi anemia subtype FA-N and predispose to childhood cancer**. *Nature genetics* 2007, **39**(2):162-164.

9. Tischkowitz MD, Sabbaghian N, Hamel N, Borgida A, Rosner C, Taherian N, Srivastava A, Holter S, Rothenmund H, Ghadirian P *et al*: **Analysis of the gene coding for the BRCA2-interacting protein PALB2 in familial and sporadic pancreatic cancer**. *Gastroenterology* 2009, **137**(3):1183-1186.

10. Vaz F, Hanenberg H, Schuster B, Barker K, Wiek C, Erven V, Neveling K, Endt D, Kesterton I, Autore F *et al*: **Mutation of the RAD51C gene in a Fanconi anemia-like disorder**. *Nature genetics* 2010, **42**(5):406-409.

11. Meindl A, Hellebrand H, Wiek C, Erven V, Wappenschmidt B, Niederacher D, Freund M, Lichtner P, Hartmann L, Schaal H *et al*: **Germline mutations in breast and ovarian cancer pedigrees establish RAD51C as a human cancer susceptibility gene**. *Nature genetics* 2010, **42**(5):410-414.

12. Xiao J, Liu CC, Chen PL, Lee WH: **RINT-1, a novel Rad50-interacting protein, participates in radiation-induced G(2)/M checkpoint control**. *The Journal of biological chemistry* 2001, **276**(9):6105-6111.

13. Kong LJ, Meloni AR, Nevins JR: **The Rb-related p130 protein controls telomere lengthening through an interaction with a Rad50-interacting protein, RINT-1**. *Molecular cell* 2006, **22**(1):63-71.

14. Park DJ, Tao K, Le Calvez-Kelm F, Nguyen-Dumont T, Robinot N, Hammet F, Odefrey F, Tsimiklis H, Teo ZL, Thingholm LB *et al*: **Rare mutations in RINT1 predispose carriers to breast and Lynch syndrome-spectrum cancers**. *Cancer discovery* 2014, **4**(7):804-815.

15. Li N, Thompson ER, Rowley SM, McInerny S, Devereux L, Goode D, Investigators L, Wong-Brown MW, Scott RJ, Trainer AH *et al*: **Reevaluation of RINT1 as a breast cancer predisposition gene**. *Breast cancer research and treatment* 2016, **159**(2):385-392.

16. Santos LS, Gomes BC, Gouveia R, Silva SN, Azevedo AP, Camacho V, Manita I, Gil OM, Ferreira TC, Limbert E *et al*: **The role of CCNH Val270Ala (rs2230641) and other nucleotide excision repair polymorphisms in individual susceptibility to well-differentiated thyroid cancer**. *Oncology reports* 2013, **30**(5):2458-2466.

17. Mantere T, Tervasmaki A, Nurmi A, Rapakko K, Kauppila S, Tang J, Schleutker J, Kallioniemi A, Hartikainen JM, Mannermaa A *et al*: **Case-control analysis of truncating mutations in DNA damage response genes connects TEX15 and FANCD2 with hereditary breast cancer susceptibility**. *Scientific reports* 2017, **7**(1):681.

18. Lhota F, Zemankova P, Kleiblova P, Soukupova J, Vocka M, Stranecky V, Janatova M, Hartmannova H, Hodanova K, Kmoch S *et al*: **Hereditary truncating mutations of DNA repair and other genes in BRCA1/BRCA2/PALB2-negatively tested breast cancer patients**. *Clin Genet* 2016, **90**(4):324-333.

19. Jagmohan-Changur S, Poikonen T, Vilkki S, Launonen V, Wikman F, Orntoft TF, Moller P, Vasen H, Tops C, Kolodner RD *et al*: **EXO1 variants occur commonly in normal population: evidence against a role in hereditary nonpolyposis colorectal cancer**. *Cancer research* 2003, **63**(1):154-158.

20. Sokolenko AP, Preobrazhenskaya EV, Aleksakhina SN, Iyevleva AG, Mitiushkina NV, Zaitseva OA, Yatsuk OS, Tiurin VI, Strelkova TN, Togo AV *et al*: **Candidate gene analysis of BRCA1/2 mutation-negative high-risk Russian breast cancer patients**. *Cancer letters* 2015, **359**(2):259-261.

21. Pepe A, West SC: **MUS81-EME2 promotes replication fork restart**. *Cell reports* 2014, **7**(4):1048-1055.

22. Faucz FR, Horvath A, Rothenbuhler A, Almeida MQ, Libe R, Raffin-Sanson ML, Bertherat J, Carraro DM, Soares FA, Molina Gde C *et al*: **Phosphodiesterase 11A (PDE11A) genetic variants may increase susceptibility to prostatic cancer**. *The Journal of clinical endocrinology and metabolism* 2011, **96**(1):E135-140.

23. Stratakis CA: **New genes and/or molecular pathways associated with adrenal hyperplasias and related adrenocortical tumors**. *Mol Cell Endocrinol* 2009, **300**(1-2):152-157.

24. Pathak A, Stewart DR, Faucz FR, Xekouki P, Bass S, Vogt A, Zhang X, Boland J, Yeager M, Loud JT *et al*: **Rare inactivating PDE11A variants associated with testicular germ cell tumors**. *Endocrine-related cancer* 2015, **22**(6):909-917.

25. Siitonen HA, Sotkasiira J, Biervliet M, Benmansour A, Capri Y, Cormier-Daire V, Crandall B, Hannula-Jouppi K, Hennekam R, Herzog D *et al*: **The mutation spectrum in RECQL4 diseases**. *European journal of human genetics : EJHG* 2009, **17**(2):151-158.

26. Petrakis NL: **Cerumen genetics and human breast cancer**. *Science* 1971, **173**(3994):347-349.

27. Hemachandar R: **Bardet-Biedl syndrome: A rare cause of end stage renal disease**. *International journal of applied & basic medical research* 2015, **5**(1):70-72.

28. Landi S, Gemignani F, Moreno V, Gioia-Patricola L, Chabrier A, Guino E, Navarro M, de Oca J, Capella G, Canzian F *et al*: **A comprehensive analysis of phase I and phase II metabolism gene polymorphisms and risk of colorectal cancer**. *Pharmacogenetics and genomics* 2005, **15**(8):535-546.

29. Bellacosa A: **Developmental disease and cancer: biological and clinical overlaps**. *American journal of medical genetics Part A* 2013, **161A**(11):2788-2796.

30. Takashima H, Boerkoel CF, John J, Saifi GM, Salih MA, Armstrong D, Mao Y, Quiocho FA, Roa BB, Nakagawa M *et al*: **Mutation of TDP1, encoding a topoisomerase I-dependent DNA damage repair enzyme, in spinocerebellar ataxia with axonal neuropathy**. *Nature genetics* 2002, **32**(2):267-272.
